# Helicobacter pylori Prevalence in Laparoscopic Sleeve Gastrectomy Specimen

**DOI:** 10.1155/2020/8843696

**Published:** 2020-12-10

**Authors:** Nada A. Sabbah, Carla Z. Saoud, Mary Deeb, Selim M. Nasser

**Affiliations:** ^1^Department of Pathology, Lebanese American University School of Medicine, Byblos, Lebanon; ^2^Lebanese American University School of Medicine, Byblos, Lebanon

## Abstract

**Introduction:**

Laparoscopic sleeve gastrectomy (LSG) has become a common surgical procedure. The value of routine histopathologic examination of the LSG specimens remains, however, a controversial issue. Helicobacter pylori was the most prevalent finding in several previous studies, but the overall results were dissimilar. We aim to assess the prevalence of Helicobacter pylori and other histopathologic findings in LSG specimens and the effect of increasing the number of sections for histology, from LSG specimens, on the rates of abnormal findings.

**Methods:**

We retrospectively reviewed the histopathologic data of all patients who had undergone LSG, in a tertiary care center, over a 4-year period (*n* = 481). Patient characteristics and histopathologic findings were recorded and analyzed.

**Results:**

Inactive chronic gastritis was the most common histopathologic finding (62.16%) followed by Helicobacter pylori gastritis (35.34%). Intestinal metaplasia was identified in 1.66% of the cases. There was no diagnosis of malignancy. Increasing the number of sections submitted for histopathologic examination resulted in a significantly higher rate of H. pylori gastritis detection.

**Conclusion:**

Routine histopathologic examination of LSG specimens may detect H. pylori in a significant proportion of patients, and increasing the number of sections for histology from LSG specimens improves the rate of detection of this bacterium and identifies individuals who may benefit from treatment.

## 1. Introduction

Laparoscopic sleeve gastrectomy (LSG) has become a common bariatric surgical procedure in many centers. This effective weight loss procedure is expected to increase as the obesity epidemic continues to grow [1]. Many pathology centers are receiving an increasing number of LSG specimens although the value of histopathologic examination of these specimens is still a matter of discussion. No standard protocol for histopathologic examination of LSG specimens has been universally adopted, and studies have reported a wide spectrum of findings. In several studies, H. pylori constituted the most significant finding, but the overall results were disparate.

We aim to assess the prevalence of H. pylori and other histopathologic findings in LSG specimens, in our center, and evaluate whether increasing the number of sections submitted for microscopic examination would increase the rates of abnormal findings.

## 2. Materials and Methods

We retrospectively retrieved 481 specimens after we systematically reviewed the histopathologic data of all patients who had undergone LSG in our center from January 2013 to December 2016. One case with a 0.8 cm gastrointestinal tumor (GIST) identified by the surgeon at the time of surgery was excluded from our series as this finding was independent of routine histopathologic evaluation. In our center, all LSG specimens are sent to the department of pathology where they are formalin-fixed then examined macroscopically, and representative sections are submitted for histology. Between 2013 and late 2014, 1 to 2 random sections per LSG specimen, measuring each 2 cm to 2.5 cm, were placed in one cassette and submitted for microscopic assessment. However, starting September 2014, and to standardize the histopathologic assessment, 4 to 6 sections, measuring each 2 cm to 2.5 cm, were placed in 3 cassettes and submitted for histology. The sections were taken, systematically, from the proximal end, distal end, and middle portion of the LSG specimens. H&E and Giemsa slides were performed and examined under light microscope, and all abnormal findings were reported. The diagnosis of inactive chronic gastritis was rendered when there was an average of 2 to 3 lymphocytes and/or plasma cells between the crypts in the lamina propria. The histopathologic findings and patients' characteristics, including preoperative endoscopies and PPI use, were recorded and analyzed. The findings in the 1 to 2 sections per LSG specimen were compared to the findings in the 4 to 6 sections per LSG specimen.

### 2.1. Statistical Analyses

Continuous data are presented as mean values with standard deviation (SD) and categorical values as frequency counts and percentages. Variables were compared by the number of sections and H. pylori diagnosis using the *χ*^2^ test for categorical variables and the Wilcoxon test for continuous values. Two-sided tests were used in all cases, and a probability threshold of 0.05 was considered significant. Variables associated with a diagnosis of H. pylori as a dependent variable were investigated using multivariate logistic regression analysis. All documented variables were evaluated independently in a univariate analysis. All data analysis was performed using IBM, SPSS statistical software Version 26.

## 3. Results

### 3.1. Sample Characteristics

The characteristics of the study population are presented in Table 1. Overall, 59.0% of patients were female, and the mean age was 40.0 ± 12.5 years. 11.2% of patients underwent preoperative endoscopy, and 10.8% reported PPI intake. One to 2 sections per specimen were submitted for microscopic examination in 221 LSG cases, and 4 to 6 sections per specimen were submitted in 260 LSG cases. Inactive chronic gastritis (60.1%) was the most common histopathologic finding followed by Helicobacter pylori gastritis (37.4%). Intestinal metaplasia was identified in 8 patients (1.7%). There was no diagnosis of malignancy, and no macroscopic lesions were identified in any of the LSG specimens. Patients' demographic characteristics and PPI intake were similar in the two groups of patients (1 to 2 sections group and 4 to 6 sections group), but the histopathologic findings and the proportion of preoperative endoscopy differed. While preoperative endoscopy is not done routinely in our center, it is performed on selected patients, based on clinical symptoms and history. 54 patients underwent preoperative endoscopy and biopsy, 42 (16.2%) patients in the 4 to 6 section group, and 12 patients (5.4%) in the 1 to 2 section group. H. pylori was identified preoperatively in 17 patients in the 4 to 6 section group and in 5 patients in the 1 to 2 section group. However, only 3 patients in the 4 to 6 section group followed an eradication course for H. pylori, and therefore, preoperative detection of H. pylori and preoperative endoscopy were inconsequential to our overall findings.

The distribution of patients' variables as a function to H. pylori and inactive gastritis diagnoses is presented in Table 2. Patients diagnosed with H. pylori tended to be older than those diagnosed with chronic gastritis (mean age 42 vs. 38), but there was no observed significant difference related to gender, PPI intake, or the fact of having had a preoperative endoscopy. However, the submission of 4 to 6 sections for microscopic examination resulted in a significantly (*p* = 0.017) higher rate of H. pylori gastritis detection (43.3%) than with the submission of 1 to 2 sections (32.6%).

Secondary multivariate analysis, performed in 469 patients, evaluated variables associated with having a diagnosis of H. pylori as opposed to inactive gastritis. In this multivariate model, increasing age, number of sections for histology (odds ratio: 1.54 [1.04–2.28]), and no intake of PPI (odds ratio: 0.49 [0.26–0.97]) were associated with having a diagnosis of H. pylori compared to inactive gastritis.

## 4. Discussion

In our series, inactive chronic gastritis was the most common finding in LSG specimens. It was detected in more than one-half of the cases (62.1%), followed by the detection of H. pylori in over one-third of the cases and by intestinal metaplasia in 1.7% of the specimens. The prevalence of inactive chronic gastritis varies widely in other studies, ranging from 13.2% to 83.4% [2–15]. This wide variation is probably due not only to different population characteristics but mainly to different criteria used for the diagnosis of chronic gastritis. Nonetheless, the diagnosis of chronic gastritis may be significant in some populations, as it may suggest the possibility of H. pylori infection that is masked by the use of proton pump inhibitors [16]. The rate of intestinal metaplasia is also variable among studies but does not exceed 3.1% [3, 5, 7, 8, 11, 12]. The rate of H. pylori in LSG specimens however varies widely [2–5, 7–14, 17–21]. It is lowest (only 1.5%) in a study from France [14] and highest (44%) in series from the Middles East [7, 17]. These disparate rates are at least partly due to different prevalence of H. pylori in the corresponding general population which fluctuates according to ethnic and socioeconomic factors and geographic locations [22–24]. It is also likely that performing an endoscopic examination and eradicating H. pylori before LSG in some centers account for lower detection of H. pylori in LSG specimens [5].

A salient finding in our study was a higher detection rate of H. pylori cases (43.3%) when 4 to 6 sections were submitted for histopathologic evaluation as compared to the submission of 1 to 2 sections (32.6% (*p* = 0.017), Table 2). This suggests that submitting systematically sections from the proximal, distal, and midportions of LSG specimens can increase the detection of cases of Helicobacter pylori gastritis and, conversely, reduce the diagnosis of chronic gastritis. In any case, centers routinely assessing LSG specimens should adopt a uniform protocol for histopathologic examination, similar to the one described by AbdullGaffar et al. [2]. There is no consensus on whether all LSG specimens should be routinely examined in pathology. Some authors consider it unnecessary unless gross pathology is identified at initial operation [2, 10, 13, 15]. Other authors highly recommend routine examination of LSG specimens that may detect early neoplastic processes or other significant findings—such as H. pylori—requiring follow-up [3, 5–7, 10, 12–14, 19]. While H. pylori in LSG specimens has not been associated with postoperative complications [9, 17, 18, 20], this microorganism is a well-established major etiological agent for peptic ulcer disease and gastric cancer [25, 26] and has been associated with the development of intestinal metaplasia that carries a potential risk for malignancy [27, 28]. In populations with high a prevalence of gastric cancer, endoscopic screening for the detection of intestinal metaplasia and H. pylori eradication have been endorsed to reduce the mortality from gastric cancer [29, 30]. Also, determining H. pylori status—along with other factors—is suggested to identify individuals with high risk for gastric cancer in low-incidence populations [30]. In our center, patients diagnosed with H. pylori gastritis on their LSG specimens undergo treatment. Therefore, finding H. pylori organisms, in more than one-third of our LSG specimens, validates our current practice of routine histopathologic examination of LSG specimens. We also suggest that routine histopathologic examination of LSG specimens would be of value in populations with known risk factors and when endoscopy is not performed preoperatively.

Our study is limited by its retrospective nature and by comparing two groups of patients over different periods of time. Arguably, our results may be flawed by an increased incidence of H. pylori over time. However, our study was done in one to two homogenous groups of the same population over sequential short periods (2013 to 2014 and 2014 to 2016) which would not allow for a significant shift in the prevalence of H. pylori. Another limitation is that our diagnostic criteria for inactive chronic gastritis is not universally adopted and may have resulted in an overestimation of its frequency. However, the latter would not affect the main findings of this study or its conclusions.

## 5. Conclusions

Routine histopathologic examination of LSG specimens may detect H. pylori in a significant proportion of patients, and increasing the number of sections for histology from different regions of LSG specimens improves the rate of detection of this bacterium and identifies individuals who may benefit from treatment.

## Figures and Tables

**Table 1:** Patients characteristics by number of sections submitted.

	Number of sections		Total	*p*
	1-2	4-6		
Number of cases	*N* = 221	*N* = 260	*N* = 481	
Age group				
15-29	51 (23.1%)	59 (22.7%)	110 (22.9%)	0.867
30-39	56 (25.3%)	75 (28.8%)	131 (27.2%)	
40-49	64 (29.0%)	68 (26.2%)	132 (27.4%)	
50-59	33 (14.9%)	35 (13.5%)	68 (14.1%)	
60-74	17 (7.7%)	23 (8.8%)	40 (8.3%)	
Age mean ± standard deviation (years)	40.4 ± 12.5	40.2 ± 12.4	40.3 ± 12.5	0.898
Sex				
Male	90 (40.7%)	107 (41.2%)	197 (41.0%)	0.924
Female	131 (59.3%)	153 (58.8%)	284 (59.0%)	
Proton pump inhibitors intake	28 (12.7%)	24 (9.2%)	52 (10.8%)	0.226
Preoperative endoscopy	12 (5.4%)	42 (16.2%)	54 (11.2%)	<0.001^∗^
Preoperative diagnosis of H. pylori	5 (2.26%)	17 (6.53%)	22 (4.57%)	+
Preoperative eradication of H. pylori	0(0%)	3(1.15%)	3(0.62%)	+
Diagnosis				0.017^∗^
Inactive gastritis	145 (65.6%)	144 (55.4%)	289 (60.1%)	
Helicobacter pylori	70 (31.7%)	110 (42.3%)	180 (37.4%)	
Intestinal metaplasia	3 (1.4%)	5 (1.9%)	8 (1.7%)	+
Reactive gastritis	2 (0.9%)	1 (0.4%)	3 (0.6%)	+
No diagnostic abnormality	1 (0.5%)	0 (0.0%)	1 (0.2%)	+

^∗^Indicates a significant relationship; +: numbers too low for statistical analysis.

**Table 2:** Patient characteristics by histopathologic findings.

	Final diagnosis		Total	*p* ^∗^
	Inactive gastritis	Helicobacter pylori		
Age group	*N* = 289	*N* = 180	*N* = 469	
15-29	87 (79.1%)	23 (20.9%)	110	<0.001^∗^
30-39	78 (60.0%)	52 (40.0%)	130	
40-49	74 (57.8%)	54 (42.2%)	128	
50-59	30 (46.9%)	34 (53.1%)	64	
60-74	20 (54.1%)	17 (45.9%)	37	
Age (years)				
Mean ± standard deviation	38.4 ± 12.4	42.6 ± 12.0	40.0 ± 12.5	<0.001^∗^
Sex				
Male	112 (59.6%)	79 (41.4%)	191 (41.0%)	0.271
Female	177 (63.7%)	101 (36.3%)	278 (59.0%)	
Preendoscopy				
Yes	26 (51.0%)	25 (49.0%)	51 (10.9%)	0.098
No	263 (62.9%)	155 (37.1%)	418 (89.1%)	
Proton pump inhibitors intake				
Yes	37 (71.2%)	15 (28.8%)	52 (10.8%)	0.134
No	252 (60.4%)	165 (39.6%)	417 (89.2%)	
Number of sections for histology				
1 to 2	145 (67.4%)	70 (32.6%)	215 (45.8%)	0.017^∗^
4 to 6	144 (56.7%)	110 (43.3%)	254 (54.2%)	

^∗^Indicates a significant relationship.

## Data Availability

Data is available upon request from the authors.

## References

[1] NCD Risk Factor Collaboration (NCD-RisC) (2017). Worldwide trends in body-mass index, underweight, overweight, and obesity from 1975 to 2016: a pooled analysis of 2416 population-based measurement studies in 128·9 million children, adolescents, and adults. *Lancet*.

[2] AbdullGaffar B., Raman L., Khamas A., AlBadri F. (2016). Should we abandon routine microscopic examination in bariatric sleeve gastrectomy specimens?. *Obesity Surgery*.

[3] Aljerian K. (2018). Histopathological findings in laparoscopic sleeve gastrectomy specimens from patients with obesity in Saudi Arabia. *Gastroenterology Research and Practice*.

[4] Almazeedi S., Al-Sabah S., Al-Mulla A. (2013). Gastric histopathologies in patients undergoing laparoscopic sleeve gastrectomies. *Obesity Surgery*.

[5] Canil A. M., Iossa A., Termine P., Caporilli D., Petrozza V., Silecchia G. (2018). Histopathology findings in patients undergoing laparoscopic sleeve gastrectomy. *Obesity Surgery*.

[6] Clapp B. (2015). Histopathologic findings in the resected specimen of a sleeve gastrectomy. *JSLS: Journal of the Society of Laparoendoscopic Surgeons*.

[7] Dogan U., Suren D., Oruc M. T. (2017). Spectrum of gastric histopathologies in morbidly obese Turkish patients undergoing laparoscopic sleeve gastrectomy. *European Review for Medical and Pharmacological Sciences*.

[8] Hansen S. K., Pottorf B. J., Hollis H. W., Rogers J. L., Husain F. A. (2017). Is it necessary to perform full pathologic review of all gastric remnants following sleeve gastrectomy?. *American Journal of Surgery*.

[9] Lauti M., Gormack S. E., Thomas J. M., Morrow J. J., Rahman H., MacCormick A. D. (2016). What does the excised stomach from sleeve gastrectomy tell us?. *Obesity Surgery*.

[10] Ohanessian S. E., Rogers A. M., Karamchandani D. M. (2016). Spectrum of gastric histopathologies in severely obese american patients undergoing sleeve gastrectomy. *Obesity Surgery*.

[11] Oner R. I., Ozdas S. (2018). Histopathological findings in morbid obese patients undergoing laparoscopic sleeve gastrectomy: does h. pylori infection effective on pathological changes?. *Obesity Surgery*.

[12] Raess P. W., Baird-Howell M., Aggarwal R., Williams N. N., Furth E. E. (2015). Vertical sleeve gastrectomy specimens have a high prevalence of unexpected histopathologic findings requiring additional clinical management. *Surgery for Obesity and Related Diseases*.

[13] Safaan T., Bashah M., El Ansari W., Karam M. (2017). Histopathological changes in laparoscopic sleeve gastrectomy specimens: prevalence, risk factors, and value of routine histopathologic examination. *Obesity Surgery*.

[14] Uguen A., Guibourg B., Badic B., Théreaux J. (2018). Reasons for pathologic examination of sleeve gastrectomy remnants in France. *American Journal of Surgery*.

[15] Komaei I., Currò G., Mento F. (2020). Gastric histopathologic findings in south Italian morbidly obese patients undergoing laparoscopic sleeve gastrectomy: is histopathologic examination of all resected gastric specimens necessary?. *Obesity Surgery*.

[16] Nasser S. C., Slim M., Nassif J. G., Nasser S. M. (2015). Influence of proton pump inhibitors on gastritis diagnosis and pathologic gastric changes. *World Journal of Gastroenterology*.

[17] Albawardi A., Almarzooqi S., Torab F. C. (2013). Helicobacter pylori in sleeve gastrectomies: prevalence and rate of complications. *International Journal of Clinical and Experimental Medicine*.

[18] Gonzalez-Heredia R., Tirado V., Patel N., Masrur M., Murphey M., Elli E. (2015). Is Helicobacter pylori associated with an increased complication rate after sleeve gastrectomy?. *Bariatric Surgical Practice and Patient Care*.

[19] Kopach P., Genega E. M., Shah S., Kim J. J., Suarez Y. (2017). The significance of histologic examination of gastrectomy specimens: a clinicopathologic study of 511 cases. *Surgery for Obesity and Related Diseases*.

[20] Shanti H., Almajali N., Al-Shamaileh T., Samarah W., Mismar A., Obeidat F. (2017). Helicobacter pylori does not affect postoperative outcomes after sleeve gastrectomy. *Obesity Surgery*.

[21] Turan G., Kocaöz S. (2019). Helicobacter pylori infection prevalence and histopathologic findings in laparoscopic sleeve gastrectomy. *Obesity Surgery*.

[22] Genta R. M., Turner K. O., Sonnenberg A. (2017). Demographic and socioeconomic influences on Helicobacter pylori gastritis and its pre-neoplastic lesions amongst US residents. *Alimentary Pharmacology & Therapeutics*.

[23] Hooi J. K. Y., Lai W. Y., Ng W. K. (2017). Global prevalence of helicobacter pylori infection: systematic review and meta-analysis. *Gastroenterology*.

[24] Leja M., Grinberga-Derica I., Bilgilier C., Steininger C. (2019). Review: epidemiology ofHelicobacter pyloriinfection. *Helicobacter*.

[25] Malfertheiner P., Megraud F., O'Morain C. (2007). Current concepts in the management of Helicobacter pylori infection: the Maastricht III Consensus Report. *Gut*.

[26] Wang C., Yuan Y., Hunt R. H. (2007). The association between Helicobacter pylori infection and early gastric cancer: a meta-analysis. *The American Journal of Gastroenterology*.

[27] Jencks D. S., Adam J. D., Borum M. L., Koh J. M., Stephen S., Doman D. B. (2018). Overview of current concepts in gastric intestinal metaplasia and gastric cancer. *Gastroenterol Hepatol (N Y)*.

[28] Polk D. B., Peek R. M. (2010). Helicobacter pylori: gastric cancer and beyond. *Nature Reviews Cancer*.

[29] Lee K. J., Inoue M., Otani T. (2006). Gastric cancer screening and subsequent risk of gastric cancer: a large-scale population-based cohort study, with a 13-year follow-up in Japan. *International Journal of Cancer*.

[30] Lin J. T. (2014). Screening of gastric cancer: who, when, and how. *Clinical Gastroenterology and Hepatology*.

